# When the protection of a threatened species depends on the economy of a foreign nation

**DOI:** 10.1371/journal.pone.0229555

**Published:** 2020-03-11

**Authors:** Daniel Fortin, Philip D. McLoughlin, Mark Hebblewhite

**Affiliations:** 1 Département de Biologie, Université Laval, Québec, Québec, Canada; 2 Department of Biology, University of Saskatchewan, Saskatoon, Saskatchewan, Canada; 3 Wildlife Biology Program, Department of Ecosystem and Conservation Sciences, W.A. Franke College of Forestry and Conservation, University of Montana, Missoula, Montana, United States of America; Kyoto University, JAPAN

## Abstract

A significant challenge of conservation biology is to preserve species in places where their critical habitat also attracts significant economic interest. The problem is compounded when species distributions occur across large spatial extents. Threatened boreal caribou (*Rangifer tarandus caribou*) epitomize this problem: their critical habitat encompasses a vast expanse of forest that also supplies much of Canada’s merchantable timber. Boreal caribou were protected under the Canada *Species at Risk Act* in 2003. We investigated putative drivers of reduced disturbance for caribou habitat since then. Where the cumulative logging footprint slowed within caribou habitat, this has resulted neither from decreases in annual allowable cut of timber nor the creation or expansion of protected areas. Rather, it has fluctuated with the American economy relative to that of Canada. For each $0.05 US lost over the $CAD, 129 km^2^ of caribou habitat was not disturbed by logging in a given year. Recent population declines have been occurring even though logging typically remained at <70% of allowed levels. Our study raises concerns about how caribou are functionally being conserved under the current application of existing legislation. In this globalized world, the economy of foreign nations is increasingly likely to govern national conservation objectives.

## Introduction

Protecting imperilled species and their critical habitat is a complex endeavor that can be costly to implement and trade off with economic development. Real and perceived socio-economic costs that come with legal obligations of protecting species frequently influence whether or not the status of a species will be recognized by conservation authorities [1]. For example, plains bison (*Bison bison bison*) were considered as Threatened by the Committee on the Status of Endangered Wildlife in Canada in 2004, but the sub-species remains unprotected in the wild under Canada’s *Species at Risk Act* (SARA) because of negative economic implications for the Canadian bison-ranching industry [2]. Literature reviews have underscored that animal species are less likely to become protected by SARA if their protection is deemed to incur economic impacts [3–6]. When it comes to the management of Canadian forests––the habitat of many species––there is a long-standing tension between the production and protection of natural resources [7] with the former generally being the favoured option [8]. Yet, once a species is listed on SARA, government agencies are legislated to put forward measures that will prevent the species from becoming extirpated, including management plans to protect critical habitat.

In Canada, conservation of boreal populations of woodland caribou (*Rangifer tarandus caribou*, hereafter boreal caribou) perhaps best epitomizes the challenge of preserving wildlife while maximizing resource extraction. The problem is compounded by the large spatial extent over which caribou are distributed. Boreal caribou are distributed in semi-sedentary populations (ranges) spread over >240 million ha, from Yukon to Labrador [9]. Human-caused rather than natural disturbances dominate the caribou ranges where the forest industry operates: areas where caribou populations are most at risk [9,10]. The oil and gas industry (especially its exploration) also has a strong impact on caribou habitat, but this impact is largely restricted to western Canada, particularly within the provinces of British Columbia and Alberta. Logging, however, occurs continent-wide across the (southern) range of boreal caribou.

Populations of boreal caribou are generally top-down driven [9]. Caribou require large areas of undisturbed boreal forest to segregate themselves from their predators and alternate prey in the system [10,11]. Human-induced disturbance conflicts with this aspect of their ecology. Timber harvesting returns mature coniferous (softwood) forest stands to an early seral stage that, once invaded by deciduous vegetation, attracts browsing ungulates [12,13]. Local increases in ungulate density can then trigger a numerical response from predator populations, which, in turn, increases predation risk to caribou [14,15]. Moreover, while wolves (*Canis lupus*) generally avoid hunting in closed coniferous forests [12,16], they take advantage of linear features such as roads (from forestry and other source) and other corridors (e.g., seismic exploration cut-lines from oil and gas exploration) [17] to venture into forests which diminishes the effectiveness of caribou habitat-selection strategies to avoid predation [18]. The response of predators like wolves to using logging-impacted habitat across boreal caribou range is also non-linear, increasing with prevalence of cutblocks on the landscape [19]. Consequently, caribou generally experience higher mortality and especially lower recruitment in areas characterized by a relatively large proportion of anthropogenic disturbance [9,20].

Preserving or protecting mature forests from disturbance is a fundamental aspect of caribou conservation planning [18,21]. Of all sources of disturbance, however, it is increasingly becoming clear that the main threat to boreal caribou is habitat alteration due to human land-use activities, most importantly the construction of linear features and timber harvest cut-blocks [9]. These two features of managed landscapes are also generally spatially associated [22], as hauling logs often requires an extensive road network. In 2012, the negative impact of cumulative disturbances including logging on vital demographic rates of boreal caribou led the Canadian government to consider that the critical habitat of caribou should be comprised of at least 65% of forests that have not been disturbed over the past 40 (by fire) or 50 (by logging) years [9].

The southern front of boreal caribou distribution has been receding for decades [23,24]. Thus, there was already a need for maintaining or even increasing protection of caribou habitat, particularly in the southern boreal, when the species became protected under SARA in 2003 [25]. General guidelines for the management of prime habitat for boreal caribou was outlined in early 2000 [e.g., 18 and references therein] with concurrent implementation in some jurisdictions, including the protection of large forest blocks within areas subject to logging activity [26]. Despite such documented actions [see also 27] it is unclear which factors have contributed to the most to the conservation of boreal caribou since the species was listed on Schedule 1 of SARA.

Across Canada, continued increases in cumulative disturbance within boreal caribou range is recognized to be largely responsible for declines in caribou populations [25,28]. Accordingly, we would expect that federal protection under SARA should have resulted in the creation of *de facto* or formalized protected areas, and, perhaps more importantly, in a reduction of habitat available to forest harvesting due to new protected areas or other constraints imposed on the logging industry, and hence a decrease in logging rates within caribou habitat from 2003 to today. However, on a national scale the Canadian forest sector is governed by macroeconomic forces. Since caribou were listed on Schedule 1 of SARA, changes in the market for Canadian wood products have also impacted on levels of forest harvesting [11,29] independently of habitat conservation actions for caribou. For example, the rise of digital alternatives to newsprint media and printing- and writing-grade paper created adverse conditions for forestry companies in the 1990s–2000s [30]. Also, in the mid-2000s, the demand for lumber increased in response to a boom in US housing, before that bubble burst in the late-2000s and the export of Canadian forest products to the US declined significantly [31]. In some ways, disconnecting market forces from habitat degradation could be a hallmark of effective conservation for such species dwelling in costly habitat to protect [32]. Here we provide a general assessment of the conflict between extinction risk for an at-risk species and resource extraction, using boreal caribou and logging activity in Canada over a 25-year period as an example.

The extent to which market forces for natural resources and related habitat degradation for an at-risk species like caribou varies, or does not vary, may be a gauge conservation policy with the latter suggesting greater effectiveness if the species is dependent on costly habitat to protect [32]. Given the strong, negative relationship between the condition of boreal caribou populations and level of forest disturbance, we investigated three factors that might contribute, directly or indirectly, to reduced disturbance of caribou habitat: 1) the creation of *de facto* or formalized protected areas; 2) the reduction of habitat available to forest harvesting; and 3) the reduction of demand for Canadian wood products by the US following economic downturns. Specifically, we began by contrasting how forest harvesting has been impacted from 1991–2015 by the annual amount of timber that could be logged on a sustainable basis (Annual Allowable Cut, AAC) versus the relative strength of the Canadian dollar vis-à-vis the US dollar (i.e., USD/CAD ratio), a macroeconomic index of these economies known to influence Canadian exports of wood products [33, see also Results section]. Bulk wood product exports tend to increase as the CAD weakens relative to the USD [33], such that exports have been relatively low during US recessions [29]. AAC and harvest of softwoods––softwood being a critical component of boreal caribou habitat [9]––were evaluated for the major jurisdictions occupied by boreal caribou. We also evaluated the covariation between Canadian exports of wood products to the US and the USD/CAD ratio. Then, restricting analysis to the area occupied by boreal caribou and logged during the period that followed its protection under SARA, i.e., 2003–2015, we contrasted the relative influence of the expansion of the Canadian protected area network versus the USD/CAD ratio. Importantly, our analysis did not imply that the USD/CAD ratio directly governed forest harvesting levels; rather, we used the ratio as a macroeconomic index of the US economy relative to that of Canada largely in terms of potential for importing and exporting wood products. A high USD/CAD tends to stimulate the export of Canadian wood products and is thus generally favourable for the Canadian forest industry [33].

## Methods

We evaluated AAC and the harvesting of softwood conifers for the provinces occupied by boreal caribou. Specifically, we extracted data on softwoods harvested between 1991–2015 in Newfoundland/Labrador, Québec, Ontario, Manitoba, Saskatchewan, Alberta, and British Columbia [34]. We extracted annual data on the categories: 1) Forest Products, softwood harvested from Provincial/Territorial/Crown Land (m^3^); and 2) Wood Supply, Softwood Potential Harvest from Provincial/Territorial/Crown Land (net merchantable m^3^), which correspond to the softwood Annual Allowable Cut (AAC). Northwest Territories was excluded from this analysis because no data were available on AAC (<0.1% of the Canadian volume of softwoods was harvested from this jurisdiction). Total softwood harvest and total AAC were estimated as the sum of all seven jurisdictions.

For 2003–2015 (post-SARA’s enactment) and within boreal caribou range, delineated based on a shape file provided by ECCCD [35], in each year we extracted the area harvested for each jurisdiction (Labrador, Québec, Ontario, Manitoba, Saskatchewan, Alberta, British Columbia, and Northwest Territories [unlike for the previous analyses, Northwest Territories could be included here because data on area harvested were available]). Area harvested was determined for each jurisdiction from data assembled by Guindon et al. [36]. Within caribou range, we identified those areas that received formal protection (total cumulative area) during this period, with information on the Canadian network of protected areas as extracted from a shape file made available by CCEA [37] for all provinces, excepting Québec. For Québec, the shape file was directly provided by MDDELCC [38]. We added data from 2015 to complement our analyses of forest harvest data. Finally, historical yearly averages of the USD/CAD ratio were extracted from FXTOP [39].

To relate dependent and independent variables, we systematically used linear regression models for data of a time series following the Autoreg procedure of SAS Institute Inc. [40] using a first-order autoregressive process, unless a second-order process (AR2) was specified (three instances). We used the exact maximum likelihood as our estimation method. We reported only the regression *R*^2^ statistic, which provides a measure of the fit of the structural part of the model after transforming for the autocorrelation, and this statistic is the *R*^2^ for the transformed regression [40]. We used regression analysis specifically to account for temporal autocorrelation; significant relationships should not be interpreted as direct causality but simply as a correlation, especially given that we use USD/CAD ratio as a macroeconomic index.

## Results

**Fig 1:**
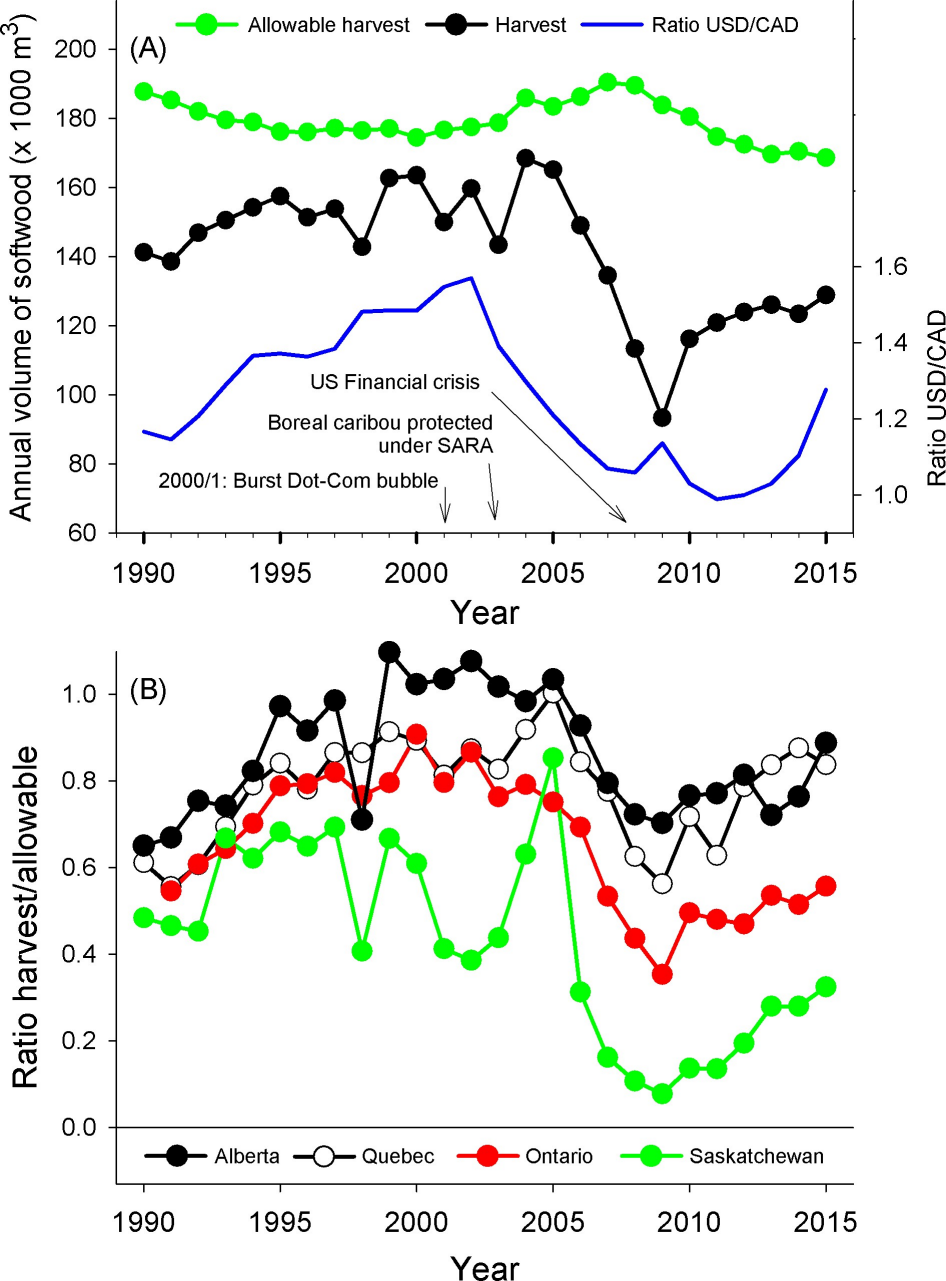
Temporal variation in allowable and observed volumes of softwood harvest, and in US/Canadian dollars. (A) Temporal variation in annual allowable volume of softwood harvest and the actual volume harvested in the main provinces where boreal caribou occur (Newfoundland/Labrador, Québec, Ontario, Manitoba, Saskatchewan, Alberta, British Columbia), together with the ratio between the US and the Canadian dollar (right axis). Years when the US Dot-Com bubble burst and the US Financial Crisis occurred, and when boreal caribou became legally protected under the Canada *Species At Risk Act* (SARA) are also indicated. (B) Examples of temporal variation in the proportion of the annual allowable softwood harvest that was actually cut in some provinces where boreal caribou occur.

Since 1991, the total volume of softwood harvested over most Canadian provinces inhabited by boreal caribou did not vary with the AAC (β [confidence limits] = -0.45 [-1.72–0.81]; *R*^2^ = 0.03; *t* = -0.75; *n* = 25 years, *P* = 0.46), but decreased as the USD weakened relative to the CAD (AR2 model; β = 40×10^6^ [11×10^6^–68×10^6^]; *R*^2^ = 0.29; *t* = 2.93; *n* = 25 years, *P* = 0.008; Fig 1A). Consistently, a larger proportion of the AAC was harvested when the strength of the USD increased with respect to the CAD (AR2 model; β = 0.38 [0.21–0.55]; *R*^2^ = 0.51; *t* = 4.66; *n* = 25 years, *P* < 0.001). On average, 67% of the AAC was harvested between 1991–2015 over all seven provinces we considered (i.e., for a given year, percent AAC harvested at the national level was estimated from the sum of the harvest volumes in all seven provinces divided by the sum of their AACs), with strong differences among jurisdictions ranging from 24–87%. Despite these variations (Fig 1B), the proportion of AAC harvested varied positively with the USD/CAD ratio in all jurisdictions, with an average *R*^2^ = 0.40 (range *R*^2^: 0.22–0.75, *P* < 0.05). During this period, the total Canadian export of wood products to the US (excluding maple products) increased with the USD/CAD ratio (β = 1.63×10^10^; *R*^2^ = 0.88; *t* = 2.18; *n* = 25 years, *P* = 0.04).

**Fig 2:**
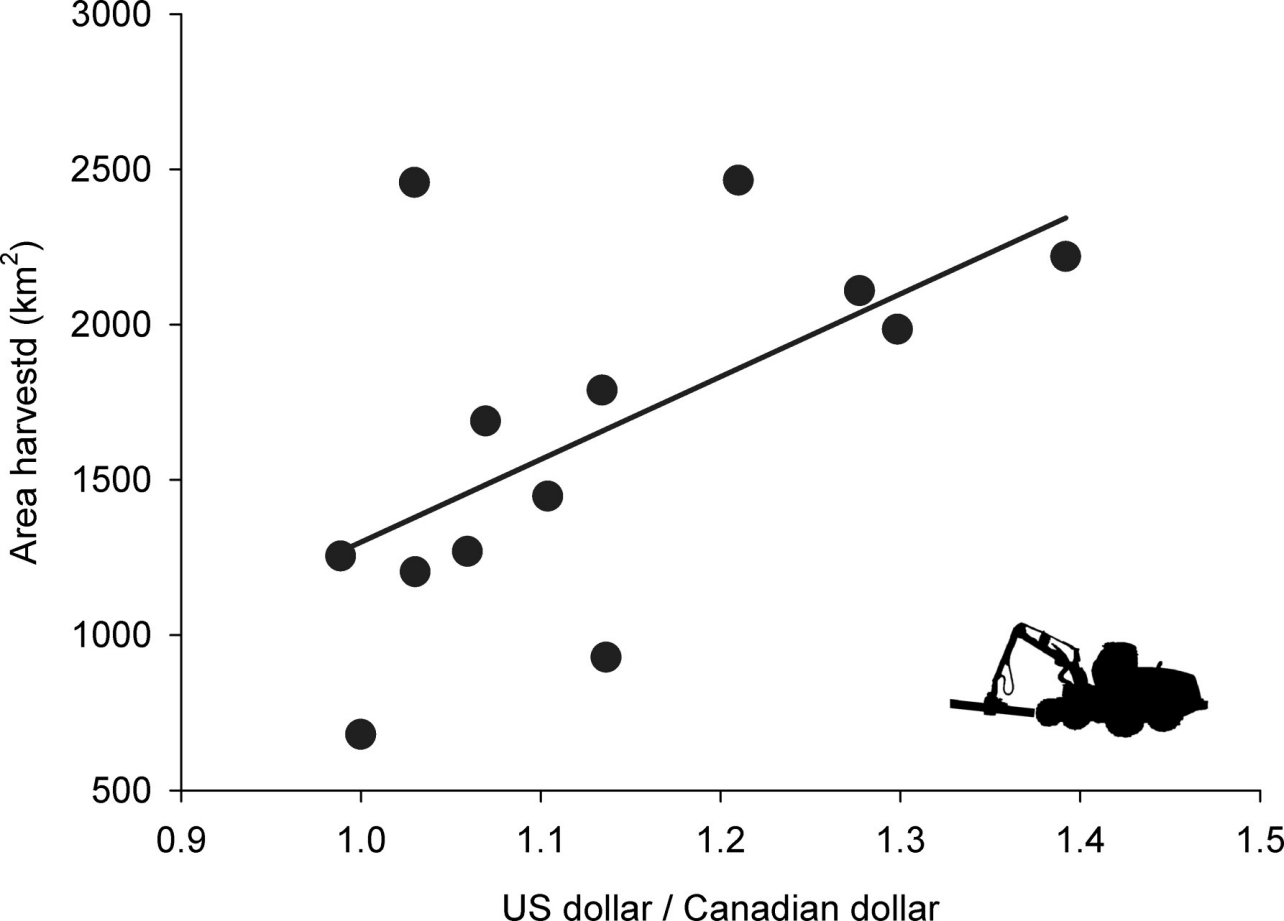
Area of forest harvested annually within boreal caribou distribution, as a function of US/Canadian dollars. The area of forest harvested annually (with 95% confidence intervals) was estimated overall for Labrador, Québec, Ontario, Manitoba, Saskatchewan, Alberta, British Columbia and Northwest Territories, specifically within the boreal caribou distribution between 2003–2015.

Since boreal caribou became protected under SARA (in 2003) until 2015, forest harvesting disturbed 1653 ± 576 km^2^ (mean ± SD) per year of caribou habitat (excluding the additional impact of roads or other linear features). During this period, annual harvest levels varied from 680–2465 km^2^, i.e., a 3.6-fold variation in annual harvest. The annual variation in the logging of caribou habitat could not be explained by the expansion of regional protected-area networks (β = -0.5 [-1.5–0.5]; *R*^2^ = 0.12; *t* = -1.19; *n* = 13 years; *P* = 0.26). Instead, annual harvest in caribou habitat gradually declined as the USD weakened relatively to the CAD (β = 2809 [538–5144]; *R*^2^ = 0.43; *t* = 2.72; *n* = 13 years; *P* = 0.02; Fig 2). We can estimate from Fig 2 that an average of 140 km^2^ of habitat remained unlogged every year for every 5 cents that the USD lost relative to the CAD––more than 26 000 football fields of caribou habitat unlogged per year.

## Discussion

We show that after more than a decade of being listed under the Canada *Species at Risk Act* (SARA), annual rates of habitat degradation for boreal caribou were better explained by the relative decline of a foreign currency––the United States––than from constraints imposed by federal Canadian or provincial legislation aimed at the animal’s protection. Habitat conservation or restoration is often viewed as a decisive step for the recovery of imperilled species [21,41], including boreal caribou [18,20]. However, here we show that the prime contributor to reduced human-caused disturbance within caribou habitat was neither a decrease in the AAC nor the establishment of new protected areas; either of which would have demonstrated a contribution of current legislation at protecting high-quality caribou habitat. Rather, we detected an indirect relationship between the US economy relative to that of Canada (i.e., USD/CAD ratio) on the logging disturbance of caribou habitat.

While the overall level disturbance of boreal caribou habitat has gradually increased since the animal became listed under the Canada *Species at Risk Act* [28], the area logged in a given year was relatively small when the US economy was relatively weak. While we expected that episodic downturns of the US economy would impact the rate of disturbance of caribou habitat in Canada (a globally significant wood-exporting nation), we were surprised by the overwhelming influence that the fortunes of the US economy had relative to Canada’s own legal measures aimed at protecting and restoring caribou habitat. We detected no apparent effect of either habitat protection or endangered species legislation on rates of caribou habitat degradation. Boreal caribou became protected under SARA shortly after the burst of the US dot-com bubble and the attacks of 9/11, two events that negatively impacted the US economy [42]. In 2008, the collapse of the housing bubble and subsequent US financial crisis led to a sharp decline in housing prices [29,43]. Because the US represents the largest export market for Canadian wood products, the demand for Canadian softwood faded as the US economy weakened, with the export of products dropping by half after boreal caribou became protected under SARA, i.e., a 55% drop between 2004 and 2012 [44]. The downturn of the US economy is reflected by the decrease in the USD/CAD ratio, such that as the USD/CAD ratio dropped, the demand in wood products decreased, as did annual rates of logging.

Softness in the Canadian timber-harvesting sector was largely caused by forces beyond the industry’s control [45]. Indeed, we found that AAC was not a strong driver of annual harvest. AAC is generally estimated for multiple years, and it is much more stable than are annual rates of logging. On average, between 1991–2015, only two-thirds of the AAC was harvested, and a higher proportion AAC was harvested under favourable market conditions, i.e., with the USD/CAD ratio being relatively high for export mainly to US markets. We do not argue that the amount of timber harvest is entirely out of government control. The fact that both international markets and domestic policies determine the role of resource production on the Canadian economy has been recognized for decades [46]. For example, the Forestry Revitalization Plan announced by the provincial government of British Columbia in 2003 ended requirements for minimum annual harvest [47]. Also, the North American Free Trade Agreement (NAFTA) removed many of the trade barriers for Canadian producers in US markets, and exports of Canadian forest products increased between 1990 and 2000 [31]. Employment in the forest sector thus can vary not only from market fluctuations but also from changes in government policies.

AAC in Canada is partly influenced by sustainability principles including the protection of biodiversity [48]. For example, AAC dropped by 20% in the province of Québec following the Coulombe commission in 2004, to meet criteria for sustainable forest management [49]. Since then, the federal government’s own progress report on implementation of the recovery plan shows continued disturbance of all caribou ranges across Canada [28]. In 2017, the Canadian government assessed changes in habitat conditions for 51 caribou populations since they published their recovery plan for boreal caribou in 2012 [28]. The comparison revealed that anthropogenic disturbances decreased during that time for 9 populations (18%), whereas disturbances increased for 29 of 51 populations (57%). This occurred even while harvest levels were only a fraction of the AAC. We might conclude that AAC, and even actual forest harvest levels, are too high to ensure the recovery of boreal caribou populations. But we might also wonder if AAC matters at all in the context of caribou conservation, given that the AAC limit is not generally reached. However, AAC could become a critical factor under favourable market conditions. This is because economic goals of all provinces and territories that harvest timber, and trading policy of governments, is to close the gap between AAC and actual amount of wood cut (e.g., [50]). Second, forest companies may push for an increase in AAC under suitable economic situations [48].

Thus far, AAC has remained fairly stable over time across Canada, in part because conservation actions to lower AAC can be offset by improvements in inventory, forest protection, and other factors that act to increase estimated sustainable harvest [51]. For example, boreal caribou conservation had a negative impact of 2% and 5% on the potential AAC of the Saguenay-Lac-Saint-Jean and the Nord-du-Québec regions of Québec for the period 2018–2023. Yet these regions still had a ‘net’ overall increase in AAC of 3.1% [52] and 3.3% [53], respectively, relative to the AAC of 2015–2018. This observation is consistent with the contention that the protection of boreal caribou should not interfere with economic growth. Indeed, there is little evidence that caribou conservation has impacted the level of timber harvest at a broad scale, with logging remaining well below AAC in most areas and most years. Québec’s premier (2014–2018) stated during his political campaign that no job would be lost for the protection of boreal caribou [54], a statement reiterated by the subsequent government, with Québec’s Minister of Forest, Wildlife and Parks stating: “We won’t lose a single job for one caribou” [55]. This statement carries much weight because individual Canadian provinces have jurisdiction over the protection of wildlife populations at risk. The federal Canadian government’s mandate is to ensure that conservation measures are sufficient to protect imperilled species. SARA still has true leverage: the federal government invoked SARA, for instance, to stop residential development in Québec in 2016 to protect the Western Chorus Frog (*Pseudacris triseriata*) [56]. Yet, it appears doubtful that the federal government intends to enforce much protection to the thousands of km^2^ of forest required to prevent further range retraction for boreal caribou.

When it comes to protecting species at risk and their critical habitat, time is of the essence. Delays in the application of law perpetuates population declines and hinders recovery efforts. Adamowicz [57] underscored frequent delays in the response of industry or responsible agencies to species recovery. Boreal caribou were already considered as threatened in 2000 [9], and 15 years later, the critical habitat necessary for their recovery has been identified, but the national and most provincial recovery plans are late [58]. More locally, for example, the Québec government recently pushed back to 2023 the application of a conservation strategy for boreal caribou [59]. When it takes decades to initiate recovery efforts there is a risk of decline for many populations, most at the southern fringe of boreal caribou range [60]. As time passes, some populations may reach a level where the allocation of resources for conservation is considered futile [61,62], leaving the area open for resource exploitation.

When conservation actions have socio-economic consequences, negotiations are likely to take place among, for example, industrial stakeholders, Indigenous groups, conservationists, and government agencies. Setting conservation targets that are ambiguous, too ambitious, or not scientifically-based will stall negotiations and delay the implementation of conservation actions [63]. Boan et al. [64] indicate that “campaigns of denial” by forestry corporations, conservative think tanks, and industry lobbyists can also result in such delays. In the specific case of boreal caribou, minimum habitat requirements for recovery have been identified from a scientific meta-analysis [9]. A typical population is predicted to face a 40% risk of declining when its range becomes disturbed by more than 35% in terms of area; however, the 35% threshold was already exceeded for 37 of the total 51 caribou populations in 2012 [9], and five years later the level of habitat degradation had increased in most provinces [28]. A problem here is that the 35% threshold has been a starting point for negotiations between the industry and governments, even though the bar is already low for recovery. Indeed, a rise in disturbance of only 5% (i.e., total of 40%) may leave the chance of self-sustainability to the flip of a coin [50%, see relationship displayed in 9].

Elected officials have the difficult task of having to protect species at risk while maintaining economic growth and employment. A conservation conundrum is that strong conservation actions can result in short-term job losses, whereas weak actions can result in unsustainable natural resource exploitation that would not stop the decline of species protected by law. Over the long-term, however, the trade-off might not be such a clear dichotomy: Lubchenco [65] pointed out that the actual choice between job and environmental conservation should be viewed in terms of short-term financial gain versus long-term, sustained prosperity. Habitat management for boreal caribou has thus far had little if any noticeable impact on AAC, and job losses in the Canadian forest industry have been more closely associated with the fluctuation of the US market than caribou conservation [64]. While the market will continue to fluctuate over time, the loss of caribou populations and the decrease in its distribution can become permanent. Indeed, attempts to reintroduce caribou to disturbed landscapes most typically fails [66].

While a number of actions were carried out to protect boreal caribou and their habitat since 2003 (e.g., deactivation or restoration of seismic lines and decommissioning roads, approval/creation of new protected areas in caribou ranges [27,28]), we could not detect any influence of those actions on overall logging rates and habitat degradation. Protecting the remaining areas of undisturbed caribou habitat will become an increasingly important conservation strategy [e.g., 67]. Many of the best remaining opportunities to establish protected areas, however, are located north of where boreal forests are allocated to timber harvesting. With climate change, those areas are expected to become more valuable to the logging industry [21], with high pressure on elected officials to allow for an expansion of industrial activities. Nevertheless, there continues to be opportunities to increase the role of protected areas for caribou recovery across the Canadian boreal forest. Canada is a signatory to the Aichi 20 under the Convention of Biological Diversity with the goal to increase protection of Canada to 17% by land area [68]. Most boreal regions of Canada are underrepresented in protected area coverage. Hence, this could be a significant opportunity to decouple globalization market forces from habitat degradation and meet the requirements for habitat protection under SARA and federal recovery strategies.

With the globalization of trade, economic fluctuations in one part of the world can affect the demography of wildlife species in another. Examples include the indirect impact of bovine spongiform encephalopathy (BSE, or ‘mad cow disease’) outbreaks in Europe on grassland bird populations in North America [69]. Here we detected a relationship between the relative strength of US economy and the degradation of boreal caribou habitat across Canada. Our study further underscores that boreal caribou populations continue to decline and habitat conditions worsen [28], even if logging levels have remained at only a fraction of what is legally allowed (i.e., harvesting remained below AAC). The situation may call into question current assessments of sustainable forest management, particularly because under the current paradigm, resource management practices should minimize the loss of ecological integrity [8] including the loss of imperiled species.

For over a decade, reductions in harvesting wood within caribou habitat in Canada were not the result of sound environmental stewardship, but rather they followed tribulations of the US economy [29], with Canadian wood product exports dropping as the USD/CAD ratio decreased [a trend already underscored, see e.g., 33]. As the export of Canadian forest products continues to expand worldwide (e.g., China and Japan are becoming major markets), however, the relationship between USD/CAD ratio and the annual level of habitat disturbance may weaken. In any case, no one expects conservation planning to rely on the relatively poor performance of trading nations. Our analysis demonstrates the crucial need to evaluate innovative economic instruments to conserve critical habitat for caribou in Canada because, to date, economic forces have driven caribou habitat disturbance.
